# The Effects of Food Limitation on Life History Tradeoffs in Pregnant Male Gulf Pipefish

**DOI:** 10.1371/journal.pone.0124147

**Published:** 2015-05-13

**Authors:** Kimberly A. Paczolt, Adam G. Jones

**Affiliations:** Department of Biology, 3258 TAMU, Texas A&M University, College Station, Texas, 77843, United States of America; Leibniz Institute for Age Research—Fritz Lipmann Institute (FLI), GERMANY

## Abstract

Syngnathid fishes (pipefishes, seahorses and seadragons) are characterized by a unique mode of paternal care in which embryos develop on or in the male’s body, often within a structure known as a brood pouch. Evidence suggests that this pouch plays a role in mediating postcopulatory sexual selection and that males have some control over the events occurring within the pouch during the pregnancy. These observations lead to the prediction that males should invest differently in broods depending on the availability of food. Here, we use the Gulf pipefish to test this prediction by monitoring growth rate and offspring survivorship during the pregnancies of males under low- or high-food conditions. Our results show that pregnant males grow less rapidly on average than non-pregnant males, and pregnant males under low-food conditions grow less than pregnant males under high-food conditions. Offspring survivorship, on the other hand, does not differ between food treatments, suggesting that male Gulf pipefish sacrifice investment in somatic growth, and thus indirectly sacrifice future reproduction, in favor of current reproduction. However, a positive relationship between number of failed eggs and male growth rate in our low-food treatments suggests that undeveloped eggs reduce the pregnancy’s overall cost to the male compared to broods containing only viable offspring.

## Introduction

Male pregnancy is a unique form of male parental care exclusively found among the pipefishes, seahorses, and seadragons of the Family Syngnathidae. In many of these species, males possess a specialized epithelial structure known as a brood pouch, and mating involves the transfer of eggs to the male’s pouch, after which the male carries the developing embryos until they emerge as independent juveniles. Male pregnancy is a complex and energetically costly physiological function that provides protection, aeration, osmoregulation, nutrition, and immune defense for the male’s developing brood [1–7]. Paternal investment during the pregnancy can be differentially allocated based on the attractiveness of females, resulting in tradeoffs between current and future reproduction [8]. The magnitude and consequences of such tradeoffs probably vary dramatically depending upon the ecological and social environment in which the pregnant male finds himself. Hence, to better understand the importance of postcopulatory brood survivorship as an evolutionary factor in pipefish, we would like to obtain a greater appreciation of how ecological factors affect the outcome of male pregnancy.

Thus far, very little empirical effort has been directed toward resolving how patterns of differential allocation in pregnant male syngnathid fishes vary based on fluctuations in ecological and population-level factors and ultimately how such relationships may affect sexual selection [9]. The goal of the present study is to investigate the effects of one such environmental variable, food availability, on the tradeoff between somatic growth and the survivorship of offspring from large, attractive females and small, unattractive females. Tradeoffs between investment in reproduction and investment in growth or condition are effectively tradeoffs between current and future reproduction because larger, healthier, or heavier individuals typically produce more offspring [10–12], a relationship previously observed in pipefish [8,13,14].

The dynamics of differential allocation in male pregnancy may be conceptually most similar to the filial cannibalism often found in nest-building fishes [15]. Both cases are characterized by taxa with indeterminate growth, exclusive male parental care, and a mechanism by which the male can recover some of the resources invested in the offspring, either by consuming eggs, as in filial cannibalism [16], or by absorbing nutrients from the eggs through the brood pouch, as has been shown in the broad-nosed pipefish [17]. A link between filial cannibalism and paternal condition has been observed in many paternally caring species, such as the sand goby [18], fantail darter [19], bluegill sunfish [20], freshwater goby [21], and threespine stickleback [22], but is not universal [23].

Here we use the Gulf pipefish (*Syngnathus scovelli*) to investigate the effects of environmental variables on paternal investment. In the Gulf pipefish, males mate with one female per brooding period, while females can mate with several males [24–26]. Once pregnant, the male carries the brood for approximately fourteen days, after which the male will mate again, usually within two days of parturition [26]. Males carry many consecutive pregnancies during the course of a single breeding season, which peaks in the summer months but lasts year-round in some populations [27]. Thus, within a breeding season, a given male may have a dozen or more pregnancies. Males spend little time between pregnancies [26], a pattern corroborated by the observation that the vast majority of males collected during the breeding season are pregnant [24,25], so if males grow during the breeding season some of this growth likely occurs while the males are pregnant. Paternal investment is also biased towards broods from large, attractive females, which exhibit increased survivorship of the eggs relative to broods from small females [8]. If male broods are energetically demanding, as multiple lines of evidence suggest [8,28], then the pregnant male faces a potential tradeoff between his own growth and the maintenance of his developing brood.

We use a two-factor experiment to examine the effects of mate size and food availability on male growth rate and offspring survivorship during pregnancy in Gulf pipefish to address two major questions. First, how do food limitation and pregnancy interact to affect male growth? Second, do males exhibit tradeoffs between somatic growth and reproduction, and does this tradeoff vary with mate attractiveness?

## Methods

Specimens were collected under Texas Parks and Wildlife permit number SPR-0808-307. This study was approved by the Institutional Animal Care and Use Committee at Texas A&M University (Animal Use Protocol #2010–108). The fish were lightly anaesthetized using clove oil prior to each measurement and sacrificed with an overdose of MS-222 at the end of the experiment.

Pregnant males and sexually mature females were collected by seine from shallow waters in the Gulf of Mexico near Aransas Pass, Texas in June and July, 2010. Fish were transported back to our live animal facility at Texas A&M University in College Station, Texas. They were housed in large coolers, which were filled with natural saltwater and aerated with portable aerators, for the five hour trip by automobile. We did not feed the fish during transit. Sexual maturity in females was evaluated by the presence of silvery-blue lateral bands, a secondary sexual trait that occurs only in mature females. Males and females were group housed in a flow-through, saltwater system and fed two-day-old enriched *Artemia* nauplii *ad libitum* until the pipefish were used in the experiment no more than fourteen days after initial capture. Pregnant males were held until they gave birth, so all males used in this experiment were not pregnant at the beginning of the experiment but had been pregnant previously, ensuring that they were reproductively active.

Experimental males were randomly assigned to one of two feeding regimes (low or high) and moved into individual 9.5 liter tanks equipped with artificial seagrass and a sponge filter. At the beginning of the trial, male total length ranged from 72 to 114 mm (mean = 90 mm). Males on the high food treatment (n = 45) were fed four times a day with two-day-old enriched *Artemia* nauplii and supplemented with copepods once per day. Males on the low food treatment (n = 45) were fed twice a day with two-day-old enriched *Artemia* nauplii. Our food treatments are relative: high food males had access to twice as much food per day, were fed more frequently, and consumed a diet of greater diversity compared to low food males. The levels of food were chosen so that high food males would always have a surplus of food while low food males would usually clear the available food between feedings. Males were maintained on this feeding regime in isolation for seven days, at which time they were randomly assigned to a mate treatment.

Males were assigned to one of three mate treatments: large female mate (n = 19, female total length 110–128 mm), small female mate (n = 38, female total length 86–103 mm), or no mate (n = 32). Larger, more active females are preferred by males [29] and broods from large females have higher offspring survivorship [8]. To keep females in good condition, each female was housed individually in a 9.5 liter tank under a high food regime during most of the day, except during her mating interactions with the male. Since female condition, and thus egg quality, may decrease over time in the lab, females were maintained in laboratory conditions for no longer than three weeks between the dates of collection and the first introduction to a nonpregnant male (trial day 1). Female size did not differ by male food treatment at any time point (Student’s t-test, trial day 1, n = 32, *P* = 0.73; pregnancy day 1, n = 32, *P* = 0.69), which confirms that females were not impacted by male feeding regimes and no unintentional bias occurred in assigning females to males in these treatments. For the mating encounters, a single female was moved into each male’s tank approximately 45 minutes after the last feeding of the day, allowed to remain with the male throughout the night, and transferred back to her own tank immediately prior to the first feeding of the next day. Male-female pairs had visual contact while separated, but were visually and chemically isolated from all other pairs. In the lab, most courtship activity occurs shortly after lights on, so this experimental design provides ample opportunity for pairs to mate. Pairs were maintained in this stage of the experiment for up to 28 days.

Mating pairs took between 1 and 21 days to mate with a mean of 7.18 days. Latency to mate did not differ between the large and small mate treatments or the high and low food treatments (two-way ANOVA, mate: F_1,32_ = 0.90, *P* = 0.35; food: F_1,32_ = 0.28, *P* = 0.60; mate*food: F_1,32_ = 31.64, *P* = 0.07). Fifteen replicates were excluded because the pair did not mate within the time allowed. Failed replicates were no more likely to occur in the low food treatment than in the high food treatment (Fisher’s exact test, *P* = 0.54). A survival analysis showed no effect of treatment on latency to mate when failed replicates were included as censored data (parametric survival analysis, Weibull distribution, chi-square = 5.56, p = 0.14). This pattern also cannot be explained by failure of these females to produce eggs during the trial, because nine of the females in the failed replicates had large, apparently mature eggs in their ovaries at the time they were dissected. Thirteen of the failed pairs were in the small female mate treatment, which may indicate that males refused to mate with these small, unattractive females as has been observed in previous experiments [8,29]. Eight more replicates were excluded from our analyses because either the male or the female died prematurely. In total, our analyses include 7 individuals in the large mate, high food treatment, 8 individuals in the large mate, low food treatment, 9 individuals in the small mate, high food treatment, and 10 individuals in the small mate, low food treatment. An additional 16 males were included in each of the control treatments (no mate, high food and no mate, low food), and these males were never given the opportunity to mate.

Male reproductive state was assessed daily, and females were removed and sacrificed the day after the male became pregnant. Pregnant males were maintained on their assigned food treatments for an additional eight days, and they were sacrificed on the ninth day of pregnancy. Offspring survivorship was then assayed by dissecting the brood pouch and counting the number of developing embryos and failed eggs. Our previous results showed that failed eggs stay in the brood pouch throughout the pregnancy, and these eggs can be easily distinguished from normal eggs midway through pregnancy [8]. Brood size is positively correlated with male length (linear regression, R^2^ = 0.12, F_1,32_ = 4.55, *P* = 0.04, b = 0.69)

To assess the success of each brood, we calculated residual offspring survivorship from a linear regression of number of surviving offspring on total number of eggs initially received by each male. Number of surviving offspring is strongly correlated with number of eggs initially received (linear regression, R^2^ = 0.92 F_1,32_ = 353.67, *P* < 0.0001), so residual offspring survivorship provides a measure of the extent to which a male has more surviving offspring than expected given his initial brood size. Positive values indicate higher than expected offspring survivorship, given the initial number of eggs, whereas negative values indicate lower survivorship than expected. One replicate was an extreme outlier in this transformation and was excluded from further analysis.

Male and female body lengths were measured to the nearest millimeter on the first day the female was added to the experiment and on the day after the pair mated. The males also were measured on the day they were added to the experiment and on the ninth day of pregnancy, which was the day the male was sacrificed. Measurements of control males mimicked those of experimental males: the “no mate” males were measured after one week on the food treatment, which coincided with the day the females were added to experimental treatments, and again nine days later, which coincided with the day a pregnant male was sacrificed. Total length was measured in both sexes.

Growth rate was calculated as the change in total length per day for the periods of time during and directly before pregnancy (Table 1). As expected, in the no mate treatment, larger males grew less than smaller males (linear regression, R^2^ = 0.32, F_1,30_ = 14.01, *P* = 0.0008); however, this pattern was not statistically significant in pregnant males (linear regression, R^2^ = 0.0748, F_1,32_ = 2.59, *P* = 0.12). To correct for the effect of size on growth rate, we standardized growth rate by taking the residual of the growth rate in pregnant males from the regression line fitted to growth rate in the control (no mate) males. Under this standardization method, males with positive standardized growth rates grew more than expected for non-pregnant males of the same size and males with negative standardized growth rates grew less than expected for non-pregnant males of the same size. We will use the term “pregnant growth rate” to apply to male standardized total length growth rate during pregnancy and “non-pregnant growth rate” to refer to male standardized total length growth rate before pregnancy.

**Table 1:** Summary of variables describing offspring survivorship, brood size, and growth rate, by treatment [mean (±S.E.)].

Treatment	Sample Size (n)	Percent Offspring Survivorship	Residual Offspring Survivorship	Brood Size	Pregnant Growth Rate, unstandardized (mm/day)	Pregnant Growth Rate, standardized (mm/day)
Large Mate, High Food	7	79.72 (± 13.5)	1.87 (± 1.02)	35.29 (± 7.04)	0.222 (± 0.042)	0.005 (± 0.039)
Large Mate, Low Food	8	98.70 (± 0.66)	2.77 (± 0.78)	31.75 (± 8.92)	0.200 (± 0.028)	-0.081 (± 0.028)
Small Mate, High Food	9	88.66 (± 4.42)	-0.18 (± 1.53)	35.11 (± 20.76)	0.194 (± 0.047)	-0.013 (± 0.042)
Small Mate, Low Food	10	76.65 (± 9.88)	-0.95 (± 1.3)	32.00 (± 16.17)	0.133 (± 0.036)	-0.106 (± 0.041)
Overall	34	85.65 (± 4.26)	0.725 (± 0.66)	33.44 (± 2.76)	0.183 (± 0.02)	-0.053 (±0.02)

We designed a multiple regression model to better understand all of the factors that may be impacting male growth rate. We included each of the following variables describing the energetic sources and sinks that may affect a pregnant male in the model as both a direct effect and an interaction effects with food treatment. Possible energetic sources include food availability (low or high) and number of failed eggs within the brood, a previously undocumented energy source suggested by the work of Sagebakken et al. [17]. Potential energy sinks include the number of successful embryos in the brood, since larger broods may make greater demands on the male. We use the number of failed eggs and number of developing embryos in this model because we assume that the benefit from egg failure and the cost of egg success will each increase incrementally as an effect on the male’s pool of available resources. However, such effects may not occur if, for example, the cost of male pregnancy is independent of the number of successful offspring. In such a case we may expect this variable to have a non-significant effect in the model. We also included latency to mate, which may act as either a source or sink. In the low food treatment, males spending more time in the experiment will have fewer stored resources available and, conversely, in the high food treatment males spending more time in the experiment will have more stored resources available. Finally, we include non-pregnant growth rate to reflect individual variation in growth. We used the lmPerm library in R (v3.0.1) for this analysis, which includes a permutation test of significance values useful when the data are non-normally distributed (in this case, latency to mate and number of failed eggs exhibit non-normality). All other statistical analyses were performed in JMP, Version 9.0 (SAS Institute, Inc.).

## Results

**Fig 1:**
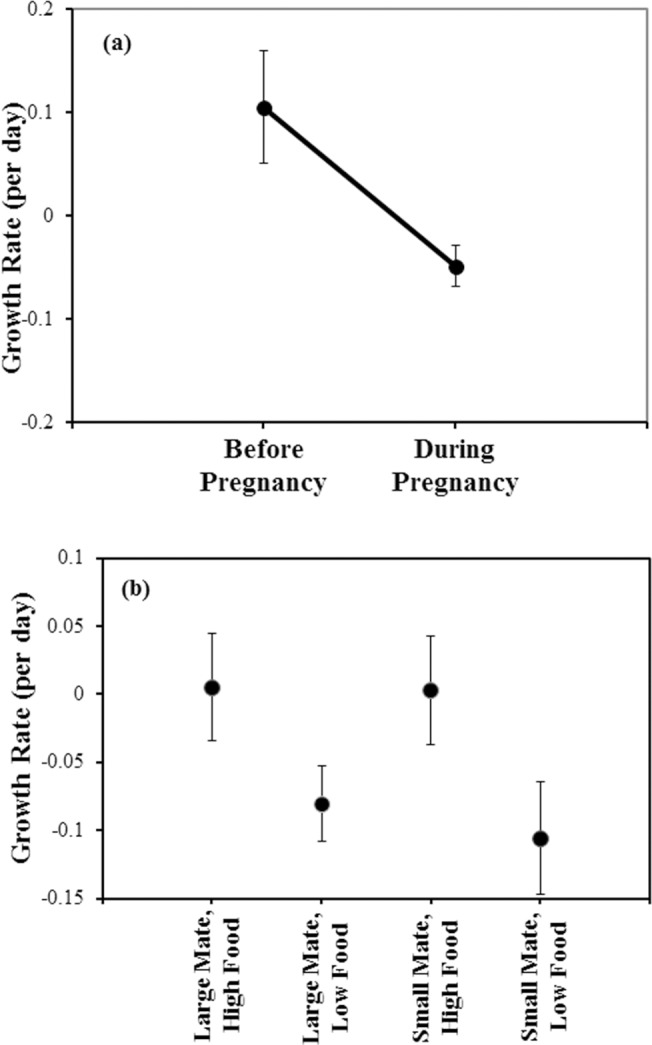
The effects on growth of pregnancy and food availability. Growth was measured before and during pregnancy and standardized to correct for the effect of male body length. Positive values indicate pregnant males grew more than non-pregnant males of the same size; negative values indicate pregnant males grew less than non-pregnant males of the same size. (a) Males grow less during pregnancy than in the time period before pregnancy (repeated measures ANOVA, within subjects, time: F_1,30_ = 5.4317, *P* = 0.0167). (b) Pregnant males on high food grow more than males on low food, regardless of mate size (two-way ANOVA, mate: F_1,32_ = 0.31, *P* = 0.58; food: F_1,32_ = 5.06, *P* = 0.03; mate*food: F_1,32_ = 0.008, *P* = 0.93).

We first investigated the effects of resource availability on growth rate. Males grew less while pregnant than in the period before pregnancy (Fig 1A, repeated measures ANOVA, within subjects, time: F_1,30_ = 5.43, *P* = 0.017). Pregnant males in high food also grew more than pregnant males in low food (Table 1, two-way ANOVA, mate: F_1,32_ = 0.31, *P* = 0.58; food: F_1,32_ = 5.06, *P* = 0.03; mate*food: F_1,32_ = 0.008, *P* = 0.93, Fig 1B, mean _all high food_ = 0.21 mm/day, mean _all low food_ = 0.16 mm/day). This result shows that our low food treatment was sufficient to cause a measurable effect.

We next used a multiple regression to investigate the effects of possible energetic sources and sinks on pregnant male growth rate (multiple regression with permutation tests: R^2^ = 0.63, *F*_9,24_ = 4.57, *P* = 0.0014). Pregnant males in the high food treatment grew more than pregnant males in the low food treatment (*P* = 0.0032). We observed no significant direct effects of non-pregnant growth rate (*P* = 0.82), latency to mate (*P* = 0.56), number of failed eggs (*P* = 0.5), and number of successful eggs (*P* = 0.19). However, the model showed significant interaction terms between food treatment and non-pregnant growth rate (*P* = 0.039), food treatment and latency to mate (*P* = 0.008), and food treatment and number of failed eggs (*P* < 0.0001) but not food treatment and number of normal embryos (*P* = 0.26). In summary, the model of growth rate shows a strong effect of food treatment as both a direct effect and an interaction term with non-pregnant growth rate, latency to mate, and number of failed embryos.

To better interpret these interaction effects, we re-ran the model of pregnant growth rate separately for the two food treatment levels. The overall model was non-significant in high food (multiple regression with permutation tests: R^2^ = 0.38, *F*_4,11_ = 1.66, *P* = 0.23, Table 2; number of failed eggs: *P* = 0.05; non-pregnant growth rate: *P* = 0.13; latency to mate: *P* = 0.27; number of normal embryos: *P* = 0.96). However, in low food (multiple regression with permutation tests: R^2^ = 0.76, *F*_4,13_ = 10.19, *P* = 0.0005, Table 2), we found that pregnant males grew more as a result of increased egg failure (*P* = 0.0006) and greater latency to mate (*P* = 0.0024), but did not vary with non-pregnant growth rate (*P* = 0.051) or number of normal embryos (*P* = 0.07). Together, these models show that the significant effects in the overall model on pregnant growth rate are driven by the low food treatment.

**Table 2:** Effects on pregnant male growth rate under low and high food treatment.

	Low Food Only ***	High Food Only
Failed eggs	0.712***	-0.550
Latency to mate	-0.664**	0.340
Successful eggs	0.298	-0.021
Non-pregnant growth rate	0.280	-0.380

Standardized regression coefficient values (*b’*) and significance level for each effect in the least square linear models of pregnant growth rate in low food (R^2^ = 0.76, *P* = 0.0005) and high food (R^2^ = 0.38, *P* = 0.23) treatments.

**P < 0.01

***P < 0.001

We next turned our attention to the pattern of resource investment exhibited by the male during pregnancy, as evidenced by offspring survivorship of the brood. Previously, we have shown that males mated to attractive, large females have higher offspring survivorship due to differential allocation of male resources across consecutive broods [8]. Our goal in this study was to see whether an environmental variable, namely food availability, impacts the pattern of differential allocation previously observed. We did find that males mated to large females had higher offspring survivorship. However, this pattern of differential allocation did not vary with resource availability (Table 1, two-way ANOVA, mate: F_1,30_ = 5.13, *P* = 0.03; food: F_1,30_ = 0.0024, *P* = 0.96; mate*food: F_1,30_ = 0.43, *P* = 0.52).

Finally, we asked whether we could recapitulate the previously observed effects of female size, brood size, and latency to mate on offspring survivorship in this independent dataset. In our previous study, broods exhibited higher offspring survivorship when males mated to larger females, carried larger broods, and mated more quickly (a shorter latency to mate) [8]. We found, as before, that offspring survivorship increased with female size and brood size and decreased with latency to mate (least squares linear regression, overall R^2^ = 0.32, F_3,28_ = 4.45, *P* = 0.01, female size, *b’* = 0.35, *P* = 0.032, brood size, Fig 1, *b’* = 0.32, *P* = 0.047, latency to mate, *b’* = -0.34, *P* = 0.037).

## Discussion

These results support the hypothesis that pregnancy is energetically costly and may indicate that pregnant males support their broods using resources that might otherwise be invested in somatic growth, a classic life-history tradeoff. A similar pattern has been observed in a related pipefish, *S*. *typhle*, where brooding males grew less than non-brooding males [28]. However, we found no effect on offspring survivorship of overall paternal resource availability or an interaction between female attractiveness and resource limitation. Taken together, these results suggest that pregnant male pipefish sacrifice investment in somatic growth, and therefore future reproduction, in favor of current reproduction.

Our statistical analysis of pregnant male growth rate further demonstrates the tradeoffs between growth and reproduction in pipefish. In the high food treatment, none of the variables in our model exhibited a significant relationship with pregnant growth rate; these males appear to have had sufficient resources to invest fully in both growth and reproduction. However, we saw a very different pattern in the low food treatment. For example, low food males that took longer to mate (high latency to mate) were consequently exposed to the low food treatment for longer and, as a result, grew less during pregnancy than low food males who mated quickly, further showing that males in the low food treatment were resource-limited.

Perhaps the most intriguing result in our model of pregnant growth rate under the low food treatment concerned the relationship between growth rate and egg failure; males with more failed eggs in their pouch appeared to grow faster than males with fewer failed eggs. Sagebakken et al. [17] showed that radioactively-labeled amino acids from the eggs were deposited in the muscle tissue of the brooding father in a related pipefish species, *S*. *typhle*. These findings provide a possible mechanism to explain the patterns we have observed here, and, together with our results, suggest that males may be able to harvest resources from the brood and then reallocate these resources for somatic growth and maintenance; however, due to our limited sample size, we cannot conclusively say that males benefit from egg failure without additional data. This pattern could also arise if pregnant males with failed eggs spend less energy supporting their brood than pregnant males without failed eggs. This pattern may be analogous to the filial cannibalism observed in many teleosts with parental care [15].

The magnitude of the effect of food availability on male growth rate observed here could easily impact male fecundity during the timeframe of a single breeding season, or even a single pregnancy. For example, males in the high food treatment grew an average of 0.21 mm/day. Hence, during the course of one pregnancy (14 days) males in this treatment grew an average of 2.94 mm. Larger males carried more offspring, with males gaining an average of 0.69 offspring for every millimeter of total length. Thus, a male in the high food treatment that grew 2.94 mm would be able to carry 2.03 additional embryos in his subsequent brood. In our low food treatment, males grew an average of 0.16 mm/day, or 2.24 mm per pregnancy, which would result in an additional 1.55 offspring in the next pregnancy. In this scenario, high food males gained 30% more offspring compared to low food males following a single reproductive cycle. Extended over just three months of the breeding season, this pattern would result in a three-embryo advantage for large males. While this difference may seem small ecologically, from a selection standpoint, it is quite large. Broods in the present study averaged 33.4 embryos, so a three-embryo advantage represents a selection differential of nearly 10 percent, which is quite strong selection. Thus, pregnant males on high food stand to gain a substantial increase in fitness compared to pregnant males on low food as a consequence of the growth discrepancy between these food treatments.

Tradeoffs between investment in parental care and growth or condition, such as we have seen here, are common across taxa with parental care but the direction of this tradeoff is highly variable. In many cases, this tradeoff negatively impacts either the parent or offspring, but not both. Our data are consistent with scenarios in which the caring parent sacrifices investment in self, via either condition or growth, in favor of reproduction (northern grass lizards [30], blue-footed booby [31], meadow vipers [32]). In other cases, the caring parent sacrifices reproduction in favor of parental condition (mountain goats [33], red-backed salamanders [34], assassin bugs [35]). Under extreme resource limitation, however, both female condition and reproduction may be impacted (white-tailed deer [36]). The magnitude of the tradeoffs between growth and reproduction may also vary within species. Increased filial cannibalism has been observed in males of smaller size classes [18,37], possibly indicating that the benefits of filial cannibalism to future reproduction is greater for smaller compared to larger individuals.

In summary, we have documented a life history tradeoff between growth and reproduction in pregnant male Gulf pipefish. We have shown that in resource-limited environments males maintain investment in their current brood, despite the costs associated with pregnancy, and instead sacrifice somatic growth, and ultimately future reproduction since brood size is limited by male body length. Furthermore, males appear to be capable of redirecting resources from failed eggs in their broods to somatic growth. These findings add to our understanding of the brood pouch as a key adaptation with far-reaching effects on the reproductive physiology and behavioral ecology of this interesting group of fishes.
